# Hot electron induced NIR detection in CdS films

**DOI:** 10.1038/srep22939

**Published:** 2016-03-11

**Authors:** Alka Sharma, Rahul Kumar, Biplab Bhattacharyya, Sudhir Husale

**Affiliations:** 1 Academy of Scientific and Innovative Research (AcSIR), National Physical Laboratory, Council of Scientific and Industrial Research, Dr. K. S. Krishnan Marg, New Delhi, 110012, India; 2 National Physical Laboratory, Council of Scientific and Industrial Research, Dr. K. S. Krishnan Marg, New Delhi, 110012, India

## Abstract

We report the use of random Au nanoislands to enhance the absorption of CdS photodetectors at wavelengths beyond its intrinsic absorption properties from visible to NIR spectrum enabling a high performance visible-NIR photodetector. The temperature dependent annealing method was employed to form random sized Au nanoparticles on CdS films. The hot electron induced NIR photo-detection shows high responsivity of ~780 mA/W for an area of ~57 μm^2^. The simulated optical response (absorption and responsivity) of Au nanoislands integrated in CdS films confirms the strong dependence of NIR sensitivity on the size and shape of Au nanoislands. The demonstration of plasmon enhanced IR sensitivity along with the cost-effective device fabrication method using CdS film enables the possibility of economical light harvesting applications which can be implemented in future technological applications.

Photodetector based devices demand broad-spectral photoresponse with very good photoresponsivity, low dark current, detectivity and should be cost effective. The next generation photodetector based devices most likely, would be made up of hybrid nanostructures improving the intrinsic properties of the base material to new and interesting functionalities. CdS is one of the cost effective materials and photodetectors made from films or nanostructures of this material have been investigated extensively for their photosensing applications^1^,^2^,^3^. The extreme sensitivity of CdS material towards visible light exhibits ~6 orders change in photoconductivity (I_photo_/I_dark_ ratio) with phototransistor properties^4^ and an ultra-fast response time was measured for nanoribbons^2^. Another intriguing feature, appealing for technology based photodetector applications using CdS material, is its cost effective fabrication process in a quick time by employing the well-established chemical bath deposition method (CBD)^5^,^6^. The broad spectral photodetection in CdS films is hampered due to intrinsic photodetection properties of the pure CdS films which respond only for wavelengths <600 nm. The photodetectors used in food analysis^7^, imaging, spectroscopy, telecommunication^8^, night survelliance^9^ etc. demand photosensitivity in the near infrared region. Presently, traditional silicon based detectors are available for NIR applications but it has large band gap (1.12 eV), responsivity in few mA range and mainly its sensitivity is limited for wavelengths >1200 nm. Since long there is a quest for simple, ultra-low cost, broad-spectral photodetectors due to miniaturization of device scale and demand for ultra-improvements in photosensitivity parameters. Taking into consideration of the technology based applications, the present research on thin films may be more appealing compared to the same with nanostructures and CdS films produced by CBD method could be one of the best options to investigate further for light harvesting applications^10^.

The present research advances on plasmonically enhanced hot electron photodetection enable materials to exhibit new optical functionalities for the wavelengths beyond their detection limit. The pioneering work of Knight *et al.* showed strong coupling between plasmons (hot electrons) and semiconductor facilitating additional optical functionality. Here, the incident NIR light (1500 nm) on metallic nanoantenna excites resonant plasmons and hot electrons injection takes place at the nanoantenna-semiconductor interface^11^. Later, Sobhani *et al.* have demonstrated plasmon-induced hot electron photocurrent generation in a grating based device patterned on a silicon substrate^12^,^13^. In another study, Wu *et al.* demonstrated the hot electron injection from Au tip into CdS nano rods and indicated the use of plasmon induced hot electron transfer for potential light harvesting applications^14^. Further photocurrent enhancements have been demonstrated recently in devices made of graphene^15^,^16^, silicon^17^, molybdenum sulphides^18^ etc. Overall, the previous literature confirms that the generation and transfer of hot electron is very feasible process at the metal semiconductor interface which can efficiently improve the photoresponsivity of the material beyond its intrinsic absorption properties.

Here we show the use of random sized Au nanoantennas generating hot electrons arising due to surface plasmon resonance phenomena and upon its decay collecting at the interface of the CdS semiconductor which eventually increases the photocurrent. We use a simple straightforward annealing temperature dependent method of random Au nano islands formation on ultra-low cost effective CdS films made by chemical bath deposition technique. The rapid annealing of thin gold film on CdS yields the formation of Au nanoislands. Different annealing temperatures resulted in random nanoisland formation having different sizes and density of particles. The absorption enhancement in CdS layer due to integration of gold nanoislands has been investigated by using FDTD software. The photoresponse of these devices is studied for visible and NIR wavelengths. The fabricated devices clearly demonstrate that the photoconductivity response shows sensitivity to visible-NIR region with high responsivity (780 mA/W), reproducibility and stability.

Figure 1 schematically depicts the device geometry utilized for photoconductivity measurements. First, a thin CdS film was deposited on the glass substrate by using chemical bath deposition technique as reported in earlier reports. Prior to CdS deposition, all the substrates were ultrasonically cleaned followed by an oxygen plasma treatment. We used the simple dc sputtering technique for the metal deposition followed by an annealing method to fabricate islands of gold on the SiO_2_ and CdS substrates. A thin layer of gold (Au) was sputtered coated on CdS film. The same sputtering chamber was used to anneal the Au films by increasing the temperature of the stage to 450°, 600° and 700 °C. The annealing temperature parameter was tuned to get the different sizes of Au nanoislands on CdS films and best photoconductivity results were obtained for 600 °C (FESEM image Fig. 1b). The size distribution of randomized Au nanoparticles sitting on the CdS film was estimated by the ImageJ software (Inset Fig. 1b). Previously, similar rapid temperature annealing method was employed to form Au nanoislands on n type silicon substrate and their size and shape dependence on annealing temperature were also observed^17^. The bright contrast of nanoparticles in Fig. 1c represents the Au nanoislands and underneath CdS film shows dark contrast. Further, additional big pads of Au/Ti were deposited by sputtering and used as electrical contacts for the photoconductivity measurements. Devices with different Au nanoisland sizes and shapes were investigated and the devices made on bare CdS film without Au nanoislands were considered as the control devices for NIR photoconductivity measurements. It is known that CdS film is not expected to absorb IR wavelengths and previous reports showed photoconductivity in visible range only^19^,^20^.

To know the photoconductive response of the CdS -Au nanoislands fabricated devices under white light illumination, we have performed current-voltage (I–V) and constant bias voltage dependent measurements (Fig. 2). First, I–V characterization of a device (Fig. 2a) shows deposited CdS films exhibit very low dark current (60 pA). The green curve indicates an intense change in photoconductivity, about 3 orders change in current under white light (halogen lamp) illumination. The blue curve shows the photoconductive response of the device for a selective (400 nm) wavelength. Figure 2b shows the spectral dependent photocurrent measurements at constant bias voltage (500 mV) and repeated cycles of light on/off states. The photocurrent (*I*_*ph*_) was calculated by subtracting the current values (*I*_*ph*_ = *I*_*light*_ − *I*_*dark*_). The stable and reproducible photocurrent response of the device was observed under different light illuminations (400, 500, 550, 600 nm). The device’s photocurrent and its dependency on the applied bias voltage for the device have been plotted in Fig. 2c. The red and yellow curves are the fits used to know the rise 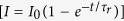 and the decay 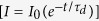 time constants respectively. The fitted curves confirm the exponential time dependency giving estimated rise and decay edges 290 and 280 ms respectively. The rise time of about millisecond^3^,^19^,^21^ or microsecond^2^,^20^ range in CdS films or nanostructures have been reported earlier. The integrated Au nanoislands-CdS films response time in millisecond are also close to the previous reported literatures. However, nanostructures exhibited better response (microsecond) but integrating them into devices are difficult thus technological success would be more if good sensitive photodetectors are demonstrated on large scale area^10^. The devices are investigated here thoroughly for their millisecond time scale photoresponse and reproducibility of the data was seen in all the measurements.

The performance of a CdS_Au nanoislands based integrated device for NIR light illumination is shown in Fig. 3. The integration of Au nanoislands in CdS films show significant change in the slope of I–V curve (Fig. 3a) when irradiated with 1064 nm light source. The IR sensitivity of these devices was characterized thoroughly by applying a constant bias voltage and exposing the device for repetitive ON/OFF cycles of NIR light source (Fig. 3b). The laser power was increased repetitively and corresponding increase in photocurrent was noticed. The rapid increase/decrease in photocurrent was observed when the light was either on/off respectively. Overall a very stable, reproducible and power dependent photocurrent response was noticed. We use above equations of exponential fits to estimate the response (red curve fit, 218 ms) and decay (yellow curve fit, 464 ms) time constants. The devices were further characterized to check the dependency of photocurrent generation on the manual increase in laser power flux density at fixed bias voltage (Fig. 3c). Over the time, the photocurrent was measured continuously and at a specific interval of time laser power was increased which is shown by the horizontal colour stripes in the Fig. 3c. The red curve shows that the manual increase in laser power increases the photocurrent and the plateau as shown by an arrow indicates that the current is saturated at that laser power. The CdS-Au nanoisland devices clearly demonstrate the photosensitivity towards small changes in incoming NIR light irradiation. Alternatively, the dependence of hot electron induced photocurrent generation on laser power density (*P*) for 1064 nm has been shown in Fig. 3d. The red curve is the simple power law fit (*I* ≈ *P*^*N*^) where exponent N determines the response of photocurrent to laser power density which we have found ≈ *0.86* i.e. close to unity and the sublinear increase in photocurrent was observed as we increase the power of the laser from 1.5 to 29 mW/cm^2^ at a constant bias voltage of 500 mV. The reason behind sublinear response could be due to the contribution in photocurrent coming from bolometeric effects and such effects have been reported in materials like carbon nanotubes^22^, graphene^23^ and metal nanoparticles.

To understand the optical properties of Au nanostructures formed on CdS films we have employed here FDTD (finite-difference time domain) method. The absorption profiles were calculated by modelling the glass (SiO_2_), CdS and Au materials. The observed simulation results (Fig. 4) show enhancement in the absorption spectra beyond the visible spectrum due to integration of Au nanoislands and results are in good agreement with the experimental photoconductivity data that demonstrated the NIR detection. The simulation results (Fig. 4a) show that absorption of NIR light in Au nanoparticles integrated with CdS film is dependent on the size and shape of Au nanoparticles. The overall maximum absorption (NIR range) was observed for Au nanoparticles with disk size of 120 nm and red shifting in the absorption profile was noticed for the sizes 80, 120, 150, 200 nm. The annealing method used here mostly forms random size nanoparticles and previously such scheme has been used experimentally and their enhancement in photoresponsivity was studied through FDTD simulations^17^. As expected from previous studies, the increase in size of the nanodisk, the red shifting in the spectra was observed. The Au nanoislands on CdS film form Schottky barrier and act as plasmon resonant antenna and the enhancement in the photoresponsivity (***R***) can be understood through the relation of Fowler function (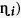 which deals with the quantum efficiency of the hot electron collection process and plasmon absorption spectrum (***S***) which is 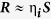 where 
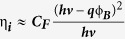
, (ϕ_**B**_ is the Schottky barrier height, ***C***_***F***_ is the Fowler emission coefficient, ***hv*** is the energy of the incident photons). The CdS films with Au electrodes junction have been studied previously and their potential barrier height is reported as ~0.76 eV^24^. To estimate the simulated photoresponsivity curve for various NIR wavelengths, we have considered above value of 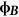. The absorption spectra shows broadband enhancement and it is known that surface plasmons of the nanoparticles are sensitive to their sizes, shape and separation distances. The process of hot electron generation and decay of the surface plasmons are wavelength dependent and we have demonstrated hot electron induced NIR photodetection for 1064 nm. The concept of hot electron transfer mechanism to study the broad band NIR photodetection was also utilized in Si films incorporated with random Au nanoislands and best responsivity was reported ~2 mA/W at 1300 nm^17^. Another study showed that nanogratings or metamaterials of metals fabricated on silicon can be used for plasmon-induced hot electron photocurrent generation and demonstrating a NIR photodetector^12^. Related to CdS material, previoulsy colloidal CdS nanorods with Au at the tip was used as a model system to study the plasmonic excitations either in CdS or Au nanoparticles and the injection of hot electrons into CdS nanorod with a quantum yield of 2.75% was achieved^14^.

The best photoresponsivity of ~780 mA/W is observed for the devices (CdS-Au nanoislands) fabricated in this study at annealing conditions 600 °C and measurements were carried out on the smaller area ~57 μm^2^ (Fig. S1 in supporting information). The increase in dark current was observed for the devices with shorter channel lengths. The devices fabricated over large area showed decrease in responsivity which could be due to efficiency of hot electron collection, grain boundaries, grain sizes and quality of CBD grown film which needs to be studied further. Note that the Au islands formed on CdS interface or at grain boundary areas in CdS film allow the absorption of subbandgap photons and generation of hot carriers. The random sized Au nanoislands sitting on the CdS film interact strongly with the incident IR light (NIR photons), absorb and transfer photon energy to the metal electrons which excite plasmon resonances at Au nanoislands (formation of surface localized plasmon excitations). The plasmons decay results into hot electrons or highly energetic carriers (photoexcited electrons) that get injected into the semiconducting CdS film (nanoislands semiconductor interface) and eventually increase the conductivity of the film through the form of photocurrent. The schematics of the FESEM image of the random Au islands formed on the CdS film was imported and used in FDTD simulation (Fig. 4c). The cross sectional image (Fig. 4d) shows the electric field enhancement in Au nanoislands formed on CdS film due to the excitation of surface plasmons and electric field localization acting further for sub-bandgap photon absorption which results in exciting the electrons of the metal i.e. generation of hot electrons. The electric field enhancement, dark red colour contrast depict the incident NIR light transmitted through the CdS films. The semiconducting CdS film actively collects hot electrons which we have observed as photocurrent.

In summary, the CdS _Au nanoisland based integrated devices demonstrate hot electron generation and their transfer in CdS films contributing to photocurrent detection. They possess very attractive optoelectronic properties such as very high responsivity (780 mA/W) and broadspectral photoresponse extending photodetection in CdS films from visible to NIR spectrum. The work on crystalline CdS nanobelt has shown ultrafast response time (~20 μs)^2^ which is far better compared to CdS thin films investigated here and thus triggers the possibility of Au nanoislands formation on CdS nanoblets for investigating ultrafast photodetection. However our work enables the potential use of Au nanoislands_CdS devices converting incident light into electrical signals which is backbone of the many photodetecting applications. Moreover, we have also used a very simple cost effective approach using glass as a substrate for CdS film deposition. The deposition of CdS films by CBD technique is well established, economical and known for an effective ultra-cost, large area production. The optimized procedure for Au nanoislands formation on CdS films is also simple and reproducible. The simplicity, high responsivity and large area fabrication make these devices more suitable candidate for ultra-low-cost Visible-NIR photodetectors. To the best of our knowledge, integrating random Au islands in CdS films and showing broadspectral (visible to NIR) region is new approach which was not attempted previously and further can be exploited in hot electron induced light harvesting or high performance photodetector applications.

## Methods

### Device Fabrication and photoconductivity measurements

The CdS films were deposited by chemical bath deposition method as described in earlier reports. The metal pads needed for the electrical connection on CdS films were sputtered coated through shadow masking procedure. The shadow masks were custom designed and made by the Tecan ltd, UK. Thin films of Au (6 nm) were also sputter coated and annealing was performed immediately after the deposition. The FESEM characterization (Zeiss, Auriga) was performed to know the Au nanoislands size distributions. The CdS-Au nanoisland devices were mounted in the probe station setup [Cascade Microtech EPS150TRIAX with shield enclosure (EPS-ACC-SE750) and triax connections] which was further custom designed for photo conductivity and low signal measurements. All the electrical measurements were carried out by Keithley 2634B source measure unit (SMU).

## Additional Information

**How to cite this article**: Sharma, A. *et al.* Hot electron induced NIR detection in CdS films. *Sci. Rep.* **6**, 22939; doi: 10.1038/srep22939 (2016).

## Supplementary Material

Supplementary Information

## Figures and Tables

**Figure 1:**
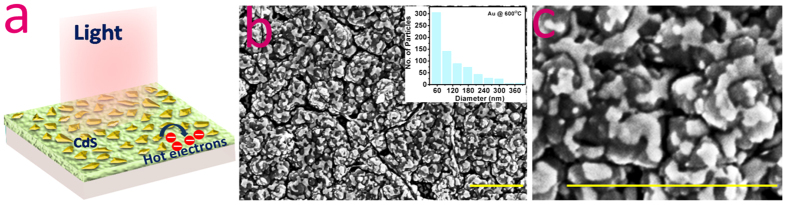
Device schematics and FESEM characterization. (**a**) The schematic representation of Au-CdS based integrated NIR photodetector and (**b**) the FESEM image of Au nanoislands formed on CdS substrate. Inset shows the particle size distributions. (**c**) The magnified image of Au nanoislands (bright contrast) easily distinguishable from the underneath CdS mat. The scale bar is 1 μm.

**Figure 2:**
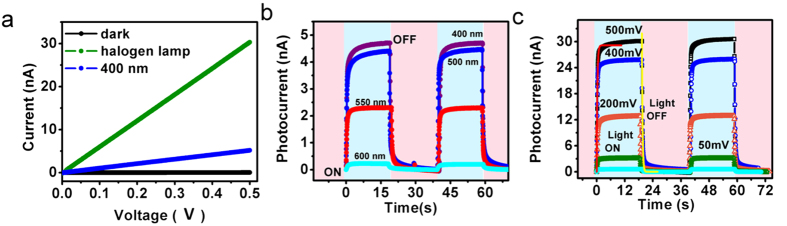
Photoconductivity measurements under white light illumination. (**a**) Current-voltage response of a representative device. The black curve represents the dark conditions i.e. the light off state. The green curve shows I–V data when halogen light was made ON and the blue curve represents the same for a particular wavelength of 400 nm. (**b**) Spectral dependent photoconductivity measurements for wavelengths 400, 500, 550 and 600 nm, at constant bias 500 mV. The labels “ON” and “OFF” represent the light on and off states, respectively. (**c**) The bias voltage dependent photoconductivity measurements performed at 50, 200, 400 and 500 mV in presence of halogen lamp.

**Figure 3:**
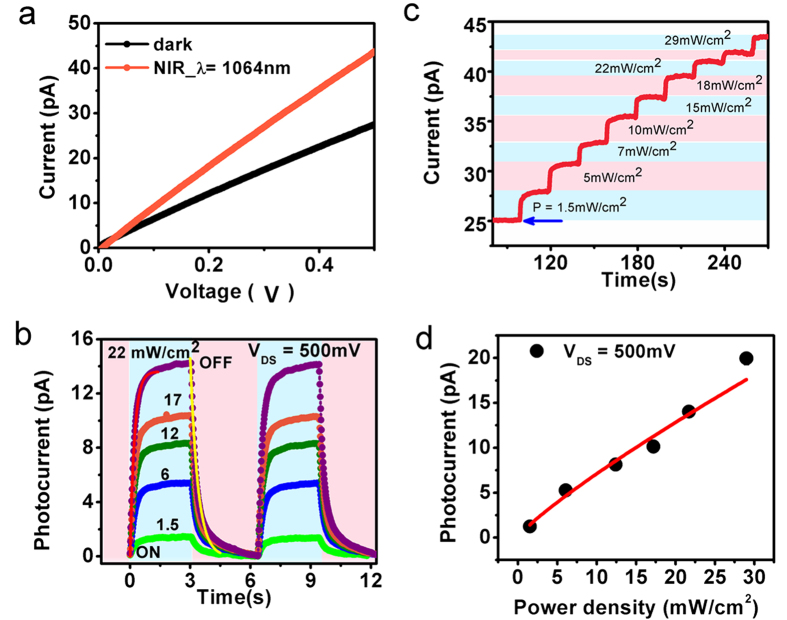
Experimental demonstration of NIR (1064 nm) detection in CdS films with Au nanoislands. (**a**) The black curve shows dark state and the orange curve shows photoresponse of the device in presence of NIR light. (**b**) Photocurrent measurement showing dependency on the illuminated laser power at constant bias voltage for repetitive on/off cycles of laser light. The red and yellow curves are the rise and decay curve fits. (**c**) The red curve depicts the response of the device monitored as a function of bias voltage, time and power of the illumining laser light. The rapid increase and saturation plateaus in current means NIR light on states and rapid increase in current saturates at a particular power of the laser as shown by the horizontal colour bar shades. (**d**) The power law dependency of photocurrent shown by the red curve fit.

**Figure 4:**
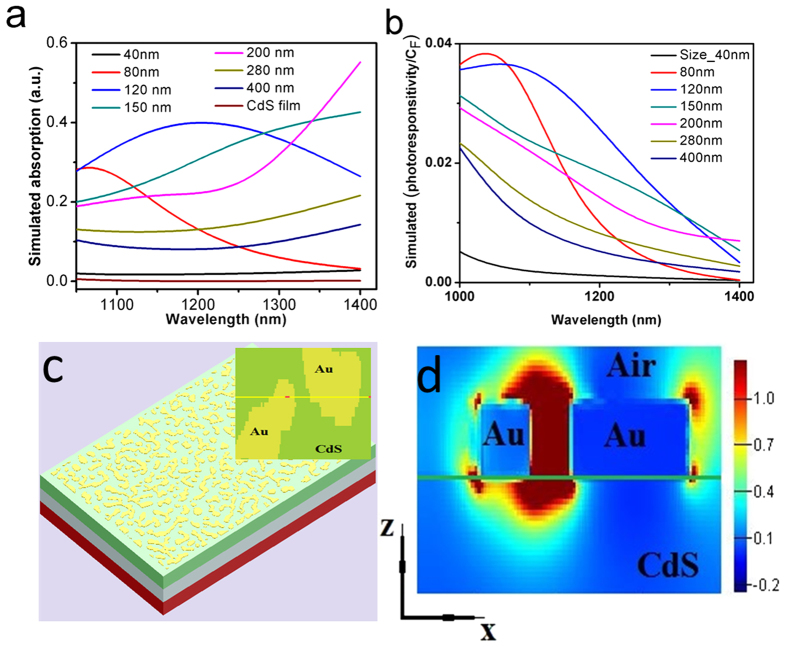
FDTD results. (**a**) The simulated absorption spectra of CdS films integrated with Au nanoislands for various wavelengths and (**b**) estimated simulated photoresponsivity. (**c**) The false colour image of FESEM micrograph exported for the visualization of total electric field distribution map in x–z plane. Inset is the magnified image showing two Au nanoislands sitting on CdS film. (**d**) The cross sectional electric field distribution map showing the confinement of the surface plasmon.
