# Supplementary material for: Hot electron induced NIR detection in CdS films
Source: Sci Rep. 2016 Mar 11;6:22939. doi: 10.1038/srep22939 (PMC4786815; doi:10.1038/srep22939)
Supplement: Supplementary Information [file srep22939-s1.pdf]

Supplementary Information

Hot electron induced NIR detection in CdS films

*Alka Sharma[1,2], Rahul Kumar[1,2], Biplab Bhattacharyya[1,2]and Sudhir Husale[1,2]**

[1]Academy of Scientific and Innovative Research (AcSIR), National Physical Laboratory, Council of Scientific and Industrial Research, Dr. K. S Krishnan Marg, New Delhi-110012, India.

[2] National Physical Laboratory, Council of Scientific and Industrial Research, Dr. K. S Krishnan Marg, New Delhi-110012, India.

**e-mail: husalesc@nplindia.org*

1. **A focused ion beam microscope was employed to deposit the platinum metal electrodes to carry out the photoconductivity measurements at shorter channel lengths**

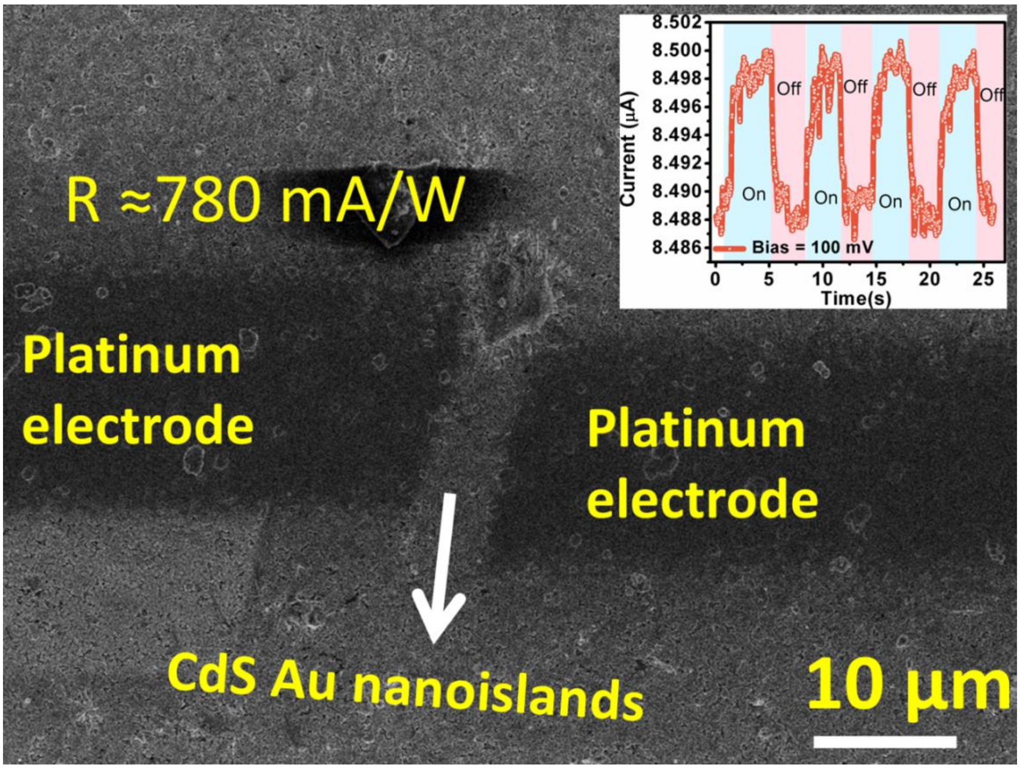

Supplementary Fig S1: Shows the Platinum electrodes deposited on CdS/glass substrate by using Focused ion beam induced metal deposition technique. Since the CdS was deposited on

insulating substrate glass, it was difficult to get the high resolution FESEM images. The area in between two pt electrodes was considered to estimate the responsivity.

2. The annealing temp 450° C , 700° C and Au nanoislands formation on CdS films

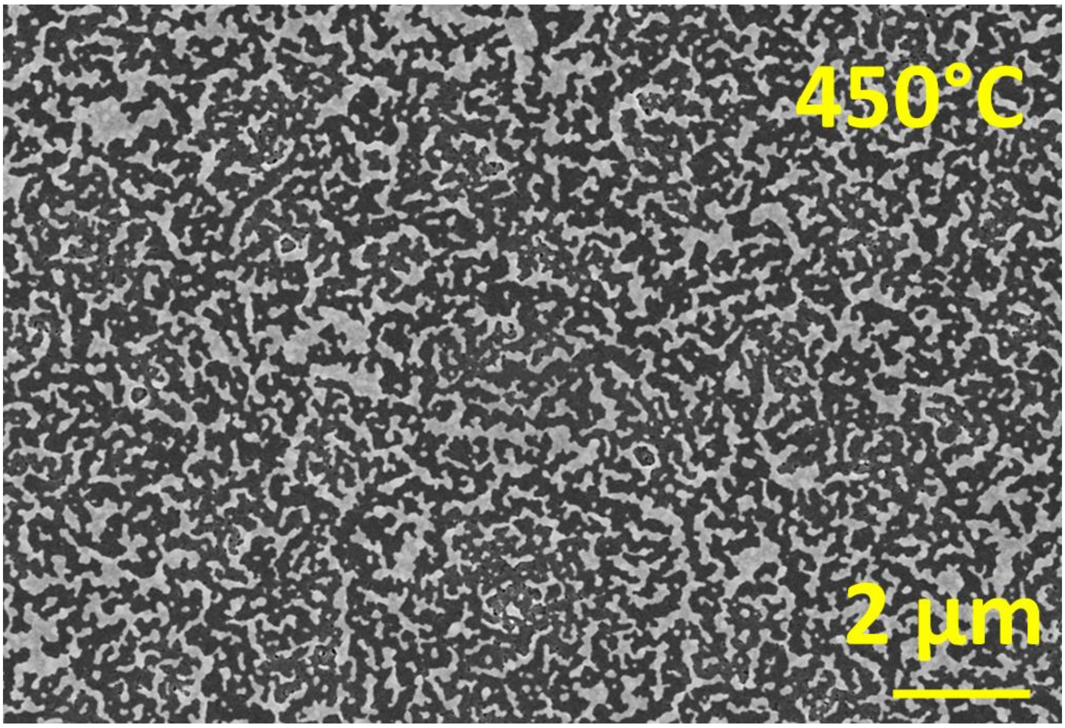

Figure S2 I A semi-continuous Au gold film was formed on CdS when annealing temp was set 450° C.

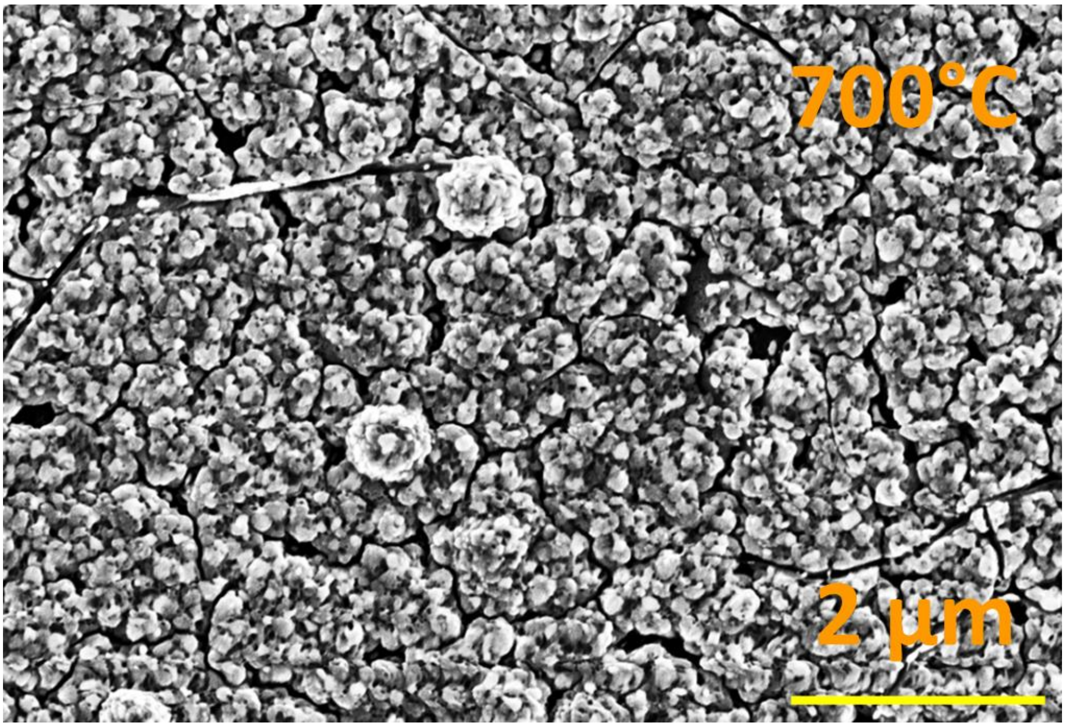

Figure S3I Nanoparticles or aggregates of Au were formed on CdS substrates when the annealing temp was 700° C.

Figure S4 I

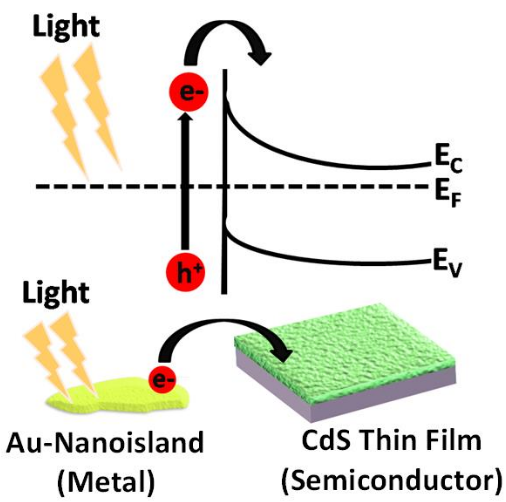

Fig S4 I Band diagram of the Au nanoisland system. The schematics show the NIR light interacting with Au nanoisland, creating holes and electrons, and transfer of generated hot carriers in the CdS film.

Basically photons coupled into metallic (Au) nanoislands excite resonant plasmons and these plasmon decays into energetic hot electrons followed by the transfer of hot electrons over potential barrier into the adjacent semiconductor material (CdS) which can generate a substantial photocurrent in our Au nanoislands CdS system. The localized enhanced field at the interface due to generation plasmons facilitates the hot electron transfer and note that

metal Au nanoparticles are extremely stable and robust system and can absorb NIR light much more efficiently compared to semiconductor CdS

Figure S5 I

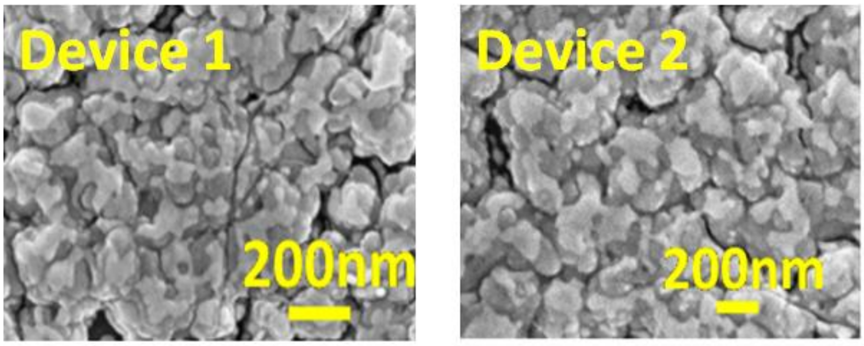

Fig S5: FESEM micrographs of the Device 1 and Device 2 (Au nanoislands formed on CdS thin films)

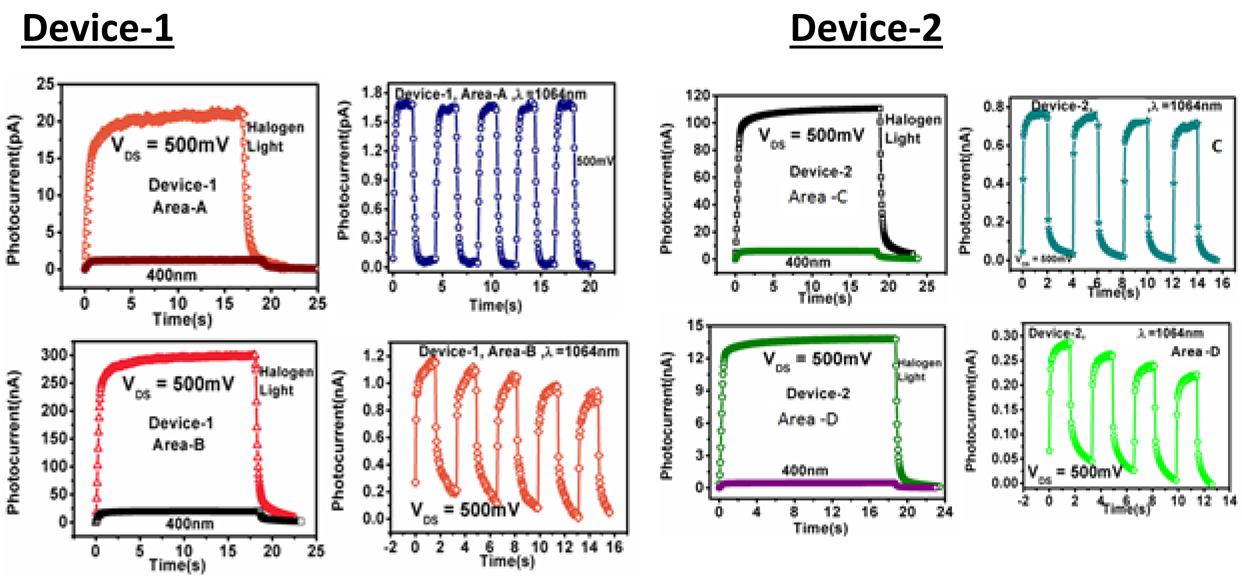

Fig S5_1: The Device_1 (left side) and Device_2 (right side) clear show the reproducibility of the data and its dependency on the wavelength and active area of photodetection.

Figure S6

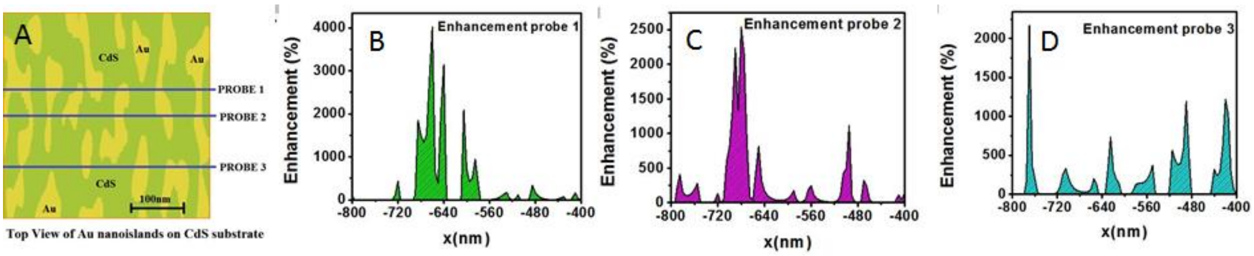

Fig S6 | Electric field enhancement when Au nanoislands formed on CdS film. A. FESEM micrograph of the device film. B. C and D represent the electric field enhancement at the probe line 1,2 and 3 respectively.

Figure S7

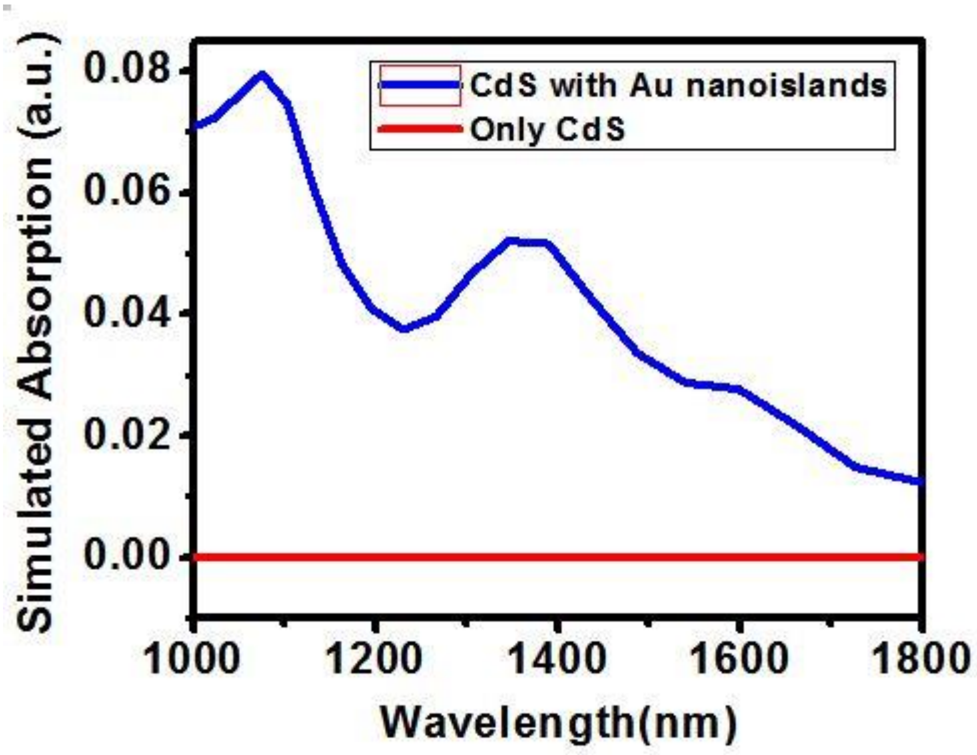

Fig S7 | Shows the absorption of NIR light by gold random nanoislands.

Table1: Comparison of the device parameters

Material	Rise time	Decay time	R (A/W)	Reference
• ZnO NW<br>• Ag NP / ZnO NW	• 320 ms<br>• 80 ms	• 3.02 s<br>• 3.27 s	---	1
CdS nanobelts	1 s	3 s	---	2
CdS film	60 ms	---	---	3
CdS NW / CdSe sheets	1.4 ms	2 ms	13.1	4
Au / CdS nanobelt	137 µs	379 µs	200	5
• ZnO NW<br>• Au nanorod / ZnO NW	• τ_1=10.9 s, τ_2=0.33 s<br>• τ_1=6.3 s, τ_2=0.238 s	• ---<br>• γ_1=16.5 s, γ_1=0.69 s	---	6
CdS nanobelts	20 µs	20 µs	7.3 x 10^4	7
Au NP / Graphene/CH3-Si nanowires array	73 µs	96 µs	1.5	8
CdS nanobelt	89 ms	31 ms	0.22	9
CdS nanoribbons	746 µs	794 µs	---	10
Monolayer MoS_2	4 s	9 s	880	11
Graphene-Bi_2Te_3 heterostructure	8.7 ms	---	35	12
PbS QD / MWCNT	9 s	---	---	13
• ZnO film<br>• ZnO film / SWNT	• 50.67 s<br>• 2.75 s	• 3.8 s<br>• 11.4 s	---	14
Au NP / ZnO NW	4.5 s	10 s	---	14
MWCNT-CuS hybrid	<0.5 s	<0.5 s	---	15
CNT / TiO_2 NW	4.3 ms	10.2 ms	---	16
Graphene / CNT	1.5 ms	---	---	17
Graphene / PbS QD	10 ms	20 ms	10^7	18
Black phosphorous	1 ms	4 ms	4.8 x 10^{-3}	19
Si-implanted β-Ga2O3 / Ti/Au electrode	18.35 s	42 s	1.45	20
Sb2Te3 film	238.7 s	203.5 s	---	21
Bi2Se3 nanosheet	0.7	1.48	20.48 x 10^{-3}	22
Bilayer MoS2	$\tau_1 = 44.5$<br>$\tau_2 = 404.7$	216.5	5.2	23
Au nanoislands formed on CdS Area 57 µm^2				Our work

References:

1. Liu, Y. et al. Ag nanoparticles@ZnO nanowire composite arrays: an absorption enhanced UV photodetector *Opt. Express*. **22**,30148-3015 (2014).
2. T. Gao, Q. H. Li, and T. H. Wang. CdS nanobelts as photoconductors. *Appl. Phys. Lett.* **86**, 173105 (2005).

3. Sharma, A et al. Channel length specific broadspectral photosensitivity of robust chemically grown CdS photodetector. *AIP Advances* **5**, 047116 (2015).
4. Li, G. et al. High-performance photodetectors and enhanced field-emission of CdS nanowire arrays on CdSe single-crystalline sheets. *J. Mater. Chem. C*, **2**, 8252 (2014).
5. Ye, Y. et al. High-Performance Single CdS Nanobelt Metal-Semiconductor Field-Effect Transistor-Based Photodetectors. *ACS Appl. Mater. Inter*. **2**(10), 2724–2727 (2010).
6. Pescaglini, A. et al. Hot-Electron Injection in Au Nanorod−ZnO Nanowire Hybrid Device for Near-Infrared Photodetection. *Nano Lett.* **14**, 6202−6209 (2014).
7. Li, L. et al. Single-Crystalline CdS Nanobelts for Excellent Field-Emitters and Ultrahigh Quantum-Efficiency Photodetectors. *Adv. Mater*. **22**, 3161-3165 (2010).
8. Luo, L.B. et al. Light trapping and surface Plasmon enhanced high-performance NIR photodetector. *Sci. Rep*. **4**, 3914 (2014).
9. Li, L. et al. Optical Sensor Based on a Single CdS Nanobelt. *Sensors* **14**, 7332-7341 (2014).
10. Jie, J.S. et al. Photoconductive Characteristics of Single-Crystal CdS Nanoribbons. *Nano Lett.* **6**(9),1887-1892 (2006).
11. Sanchez, O.L. et al. Ultrasensitive photodetectors based on monolayer MoS_2. *Nat.Nanotechnol.* **8** (2013).
12. Qiao, H. et al. Broadband Photodetectors Based on Graphene-Bi_2Te_3 Heterostructure. *ACS Nano*. **9**(2),1886–1894 (2015).
13. Feng, W. et al. A layer-nanostructured assembly of PbS quantum dot/multiwalled carbon nanotube for a high-performance photoswitch. *Sci. Rep.* **4**, 3777 (2014).
14. Chang, J. et al. High-performance photoresponse from single-walled carbon nanotube–zinc oxide heterojunctions. *J. Phys. D: Appl. Phys.* **44**, (2011).
15. Zhan, Z. et al. Photoresponse of multi-walled carbon nanotube–copper sulphide (MWNT–CuS) hybrid nanostructures. *Phys. Chem. Chem. Phys*. 13, 20471–20475 (2011).
16. Hsu, C.Y. et al. Supersensitive, Ultrafast, and Broad-Band Light-Harvesting Scheme Employing Carbon Nanotube/TiO_2 CoreShell Nanowire Geometry. *ACS Nano.* **6**(8), 6687–6692 (2012).
17. Lu, R. et al. High Photoresponse in Hybrid Graphene−Carbon Nanotube Infrared Detectors. *ACS Appl. Mater. Interfaces* **5**, 11703−11707 (2013).
18. Konstantatos, G. et al. Hybrid graphene–quantum dot phototransistorswith ultrahigh gain. *Nat. Nanotechnol.* **7**, 363–368 (2012).
19. Huan Zhao ,Qiushi Guo,Fengnian Xia and Han Wang Two-dimensional materials for nanophotonics application. *Nanophotonics* **4**,128–142 (2015).
20. Oh, S. et al. Development of solar-blind photodetectors based on Si-implanted β-Ga_2O_3. *Opt. Express.***23**(22), 28300-28305 (2015).
21. Zheng, K. et al. Optoelectronic characteristics of a near infrared light photodetector based on a topological insulator Sb_2Te_3 film, *J. Mater. Chem. C* **3**, 9154-9160 (2015).
22. Zang, C. et al. Photoresponse properties of ultrathin Bi_2Se_3 nanosheets synthesized by hydrothermal intercalation and exfoliation route. *Appl. Surf. Sci.* **316**, 341-347 (2014).
23. Wang, W. et al. Hot Electron-Based Near-Infrared Photodetection Using Bilayer MoS_2, *Nano Lett*. **15**, 7440−7444 (2015).

Table 2: Device's active surface area and response time

S.No.	Device Name	Area (μm^2)	Light Source Power density (mW/cm^2)	I_{ph}(nA) I_{ph}-1	I_{ph}-2	R(mA/W) R-1	R-2	Rise Time (ms)	Decay Time (ms)
1.	Device-1	L= 95µm W= 85µm A(A)= $8075 x 10^{-12} m^2$	400nm P_d:0.30mW/cm^2	0.856	1.48	35.33	61.0	389	885
			NIR(1064nm) P_d:29mW/cm^2	1.60	1.70	0.683	0.725	175	210
		L= 80µm W= 65µm A(B)= $5200 x 10^{-12} m^2$	400nm P_d:0.30mW/cm^2	12.28	21.0	787	1320	363	525
			NIR(1064nm) P_d:29mW/cm^2	1.15	0.88	0.762	0.583	72	170
2.	Device-2	L= 65µm W= 100µm A(A)= $6500 x 10^{-12} m^2$	400nm P_d:0.30mW/cm^2	4.30	6.77	220	347	364	543
			NIR(1064nm) P_d:29mW/cm^2	0.687	0.765	0.362	0.405	57	185
		L= 130µm W= 180µm A(B)= $23400 x 10^{-12} m^2$	400nm P_d:0.30mW/cm^2	0.402	0.759	5.73	10.68	315	445
			NIR(1064nm) P_d:29mW/cm^2	0.259	0.217	0.0382	0.0320	63	280
3.	Device-3	**FIB Fabricated Device** L=5.2um W= 11um Area =57.2um^2	NIR(1064nm) P_d:29mW/cm^2	12.62	13.01		**780**	68	186
